# Development of key indicators to quantify the health impacts of climate change on Canadians

**DOI:** 10.1007/s00038-013-0499-5

**Published:** 2013-07-30

**Authors:** June J. Cheng, Peter Berry

**Affiliations:** 1 Public Health and Preventive Medicine Residency, Department of Clinical Epidemiology and Biostatistics, McMaster University, Hamilton, ON Canada; 2 Climate Change and Health Office, Health Canada, Ottawa, ON Canada

**Keywords:** Climate change, Health surveillance, Indicator, Mortality, Morbidity, Public health

## Abstract

**Objectives:**

This study aimed at developing a list of key human health indicators for quantifying the health impacts of climate change in Canada.

**Methods:**

A literature review was conducted in OVID Medline to identify health morbidity and mortality indicators currently used to quantify climate change impacts. Public health frameworks and other studies of climate change indicators were reviewed to identify criteria with which to evaluate the list of proposed key indicators and a rating scale was developed. Total scores for each indicator were calculated based on the rating scale.

**Results:**

A total of 77 health indicators were identified from the literature. After evaluation using the chosen criteria, 8 indicators were identified as the best for use. They include excess daily all-cause mortality due to heat, premature deaths due to air pollution (ozone and particulate matter 2.5), preventable deaths from climate change, disability-adjusted life years lost from climate change, daily all-cause mortality, daily non-accidental mortality, West Nile Disease incidence, and Lyme borreliosis incidence.

**Conclusions:**

There is need for further data and research related to health effect quantification in the area of climate change.

**Electronic supplementary material:**

The online version of this article (doi:10.1007/s00038-013-0499-5) contains supplementary material, which is available to authorized users.

## Introduction

**Fig. 1:**
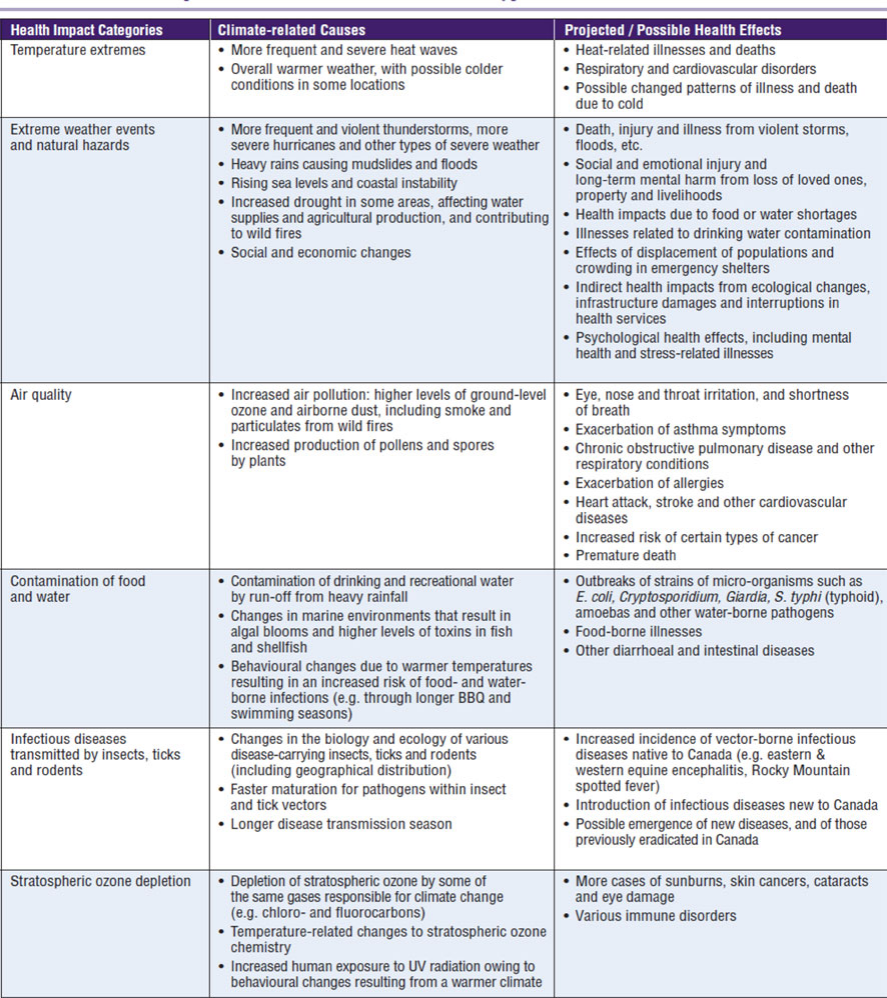
Health impacts of climate change on Canadians (reproduced from Seguin 2008)

Climate change is receiving increasing attention as a multifarious driver of a variety of negative health impacts. Climate may impact health through, for instance, temperature changes, extreme weather events, air pollution, and through the exacerbation of food shortages, and vector-, food-, and water-borne diseases. Climate change may also impact health through the knock-on effects of human migration and socioeconomic disruption (Patz et al. 2000, 2001, 2005; McMichael et al. 2004; Ebi et al. 2006; IPCC 2007; O’Neill and Ebi 2009). In Canada, a comprehensive 2008 report by Seguin discusses the health impacts most relevant to Canadians (Fig. 1).

Health authorities in Canada require information about risks to health from climate variability and change to be able to undertake needed adaptation measures (Clarke and Berry 2011). Climate change and health vulnerability assessments can provide this information and guidance is available to health authorities for undertaking these studies (Ebi et al. 2012; Health Canada 2011). Critical to these assessments are data on health outcomes that may result from climate-related exposures; such data can be measurable and quantifiable by human mortality and morbidity indicators. Climate change health outcome indicators support efforts to protect health by offering the following applications:

- the provision of accurate, quantified data for assessing human health vulnerability to climate change;
- the monitoring of climate change health effects by local, provincial/territorial, and federal governments;
- the projection of climate-related disease burdens for public health planning and intervention;
- the evaluation of the effectiveness of public health adaptations, and;
- the provision of a standardized language for describing climate change health effects across different sectors.

There have been several efforts to develop indicators for quantifying the health impacts of climate change. In the United States, a State Environmental Health Indicators Collaborative established by the Council of State and Territorial Epidemiologists put forward recommendations for climate change health indicators (English et al. 2009). A symposium held by the World Health Organization (WHO) and the European Center for Environment and Health identified eight health-relevant climate change indicators that include human mortality indicators and other indicators such as air quality and flooding (Dalbokova et al. 2009 as referenced by English et al. No date). In addition, larger lists of climate change indicators that are not restricted to health have been developed in the recent years (National Research Council 2010; California EPA 2009, and DARA 2012; US EPA 2012). Recently, a database on climate and health related indicators became publicly available through the Metadata Access Tool for Climate and Health (MATCH) (United States Global Change Research Program 2013). Many of these reports propose the adoption of indicators of exposure to climate events and hazards such as the number of extreme heat days, number of floods, and the prevalence of infectious disease vectors. Guided by the proposed uses for the indicators in Canada, we focus on the measurement of health outcome trends (i.e., mortality and morbidity) associated with climate-related hazards and not on broader measures of vulnerability (e.g., indicators of exposure, adaptive capacity).

Currently, there are no Canadian studies that have attempted to compile a set or “basket” of key climate-related health outcome indicators on a national scale. However, in Quebec, indicators for this province have been developed, for example, for health problems related to heat health disorders, heavy precipitation, floods, landslides, drought, strong winds, lightening, and forest fires (Tairou et al. 2010a, b, c; Bustinza et al. 2010a, b; Bélanger et al. 2010). This paper applies a structured method for evaluating the suitability of existing health indicators for use anywhere in Canada. It is anticipated that the methods used in this report will be applicable and transferrable to other countries and regions looking to undertake similar work.

## Methods

OVID Medline was searched in December 2012 using key terms “climate change, change, climate, global, global warming, greenhouse effect, health, health indicator*, health status indicators, health surveillance, health surveys, indicator*, morbidity, mortality, population surveillance, surveillance, vital statistics, and warming.” A total of 496 article abstracts were reviewed. Where appropriate, the entire article was reviewed for more detailed information. Inclusion criteria were as follows:

- the article must include the words “climate change” or “global warming” in the body of its text;
- the article must discuss human health in a quantitative fashion using indicators;
- the article must make implications to suggest that climate change can affect human health or it must note the link between climate and health;
- the article must be relevant to the developed-world context;
- the article must be in English, and;
- the article must be retrievable electronically.

Drawing upon this literature review, we developed a list of indicators that have been used to measure climate-related health outcomes (Online Resource). We then reviewed several public health frameworks and other studies of climate change indicators to identify criteria by which to evaluate the list of indicators for use in the Canadian context (Online Resource). A rating scale based on the selected criteria was developed to evaluate each indicator and all indicators were rated. Given the core objective of developing indicators that adequately capture the impacts of climate change on health, the criteria “specificity” which describes the linkage between the health outcome and climate change as a causal factor was weighted more heavily than others. The score from specificity was multiplied by two and added to other scores to calculate the total score for each indicator. If a candidate indicator was less specific to climate change effects due to other known important influencing factors, it was considered less desirable for inclusion in the final “basket” of indicators.

## Results

A total of 77 existing climate change and health outcome indicators were identified in the literature review. These indicators were evaluated by the following criteria:

- Specificity.
- Availability/feasibility.
- Quality.
- Comparability over time and place.
- Relevance to planning.

The definitions for these criteria can be found in detail in Table 1. The scoring assignments for each of the above evaluation criteria are described in Table 2. Tables 3, 4, 5, 6, 7, 8, 9 contain the actual, detailed scoring results for each indicator and Table 10 provides a summary of the total scores of all 77 indicators by descending order.

**Table 1:** Criteria definitions for evaluating human health indicators

Criteria	Definition
Specific	Responds to changes in climate and less sensitive to alternate explanations
Availability/feasibility	Readily available for areas and time periods required. There are no unreasonable obstacles or constraints on access, and the information can be used without restrictions. For modeled indicators, the modeling process is either completed or easily understood by those without specialized training.
Quality	Collected in a predictable and consistent manner using reliable methods. Data integrity is maintained in storage, management, and manipulation. Accuracy is monitored through regular audits and results are reported. If under- or over- reporting is present, this is noted or corrected for.
Comparability over time and place	Can be compared over time and with other geographic areas, standards or benchmarks. The information must be recorded and reported in like manner over time and place.
Relevance to planning	Provides information that advances the understanding of population health and can be used to monitor health

**Table 2:** Climate change and health outcome indicator rating scale

Indicator criteria	Score = 0	Score = 1	Score = 2
Specificity	Indicator measures health outcome relevant to climate change; but there are other stronger influences from factors outside of climate	Indicator measures health outcome related to climate change, but there are other but less important influences from factors outside of climate	Indicator measures health outcome related to climate change, and this health outcome is mostly not vulnerable to other influencing factors aside from climate
Availability/feasibility	Data is not available within a reasonable time frame (i.e. 1 year). For modeled outcomes, the indicator is not easily calculated without specialized training	Restricted data access to local health authorities, or is not frequently available (i.e. only every few years). For modeled outcomes, specific calculation methods are available	Data accessible to local health authorities, and are frequently available at needed intervals. For modeled outcomes, data is available in modeled, completed form
Quality	Unknown quality or known major quality concerns	Known minor quality concerns	High quality
Comparability over time and place	Indicator is not comparable over time or population groups, i.e. not calculated the same way.	Indicator is comparable over time and some other population groups (i.e. has some methodological inconsistencies)	Indicator is comparable to other time periods as well as other population groups (including other provinces, countries, or international data)
Relevance to planning	Indicator is not important to the population group; for some infectious diseases, this means there are no current threats of such diseases in Canada	Indicator is important to a vulnerable portion of the population group	Indicator is important to most members of the population group

**Table 3:** Scoring of existing climate change and health indicators for temperature-extremes related health effects

Indicator	Specificity (weighted × 2)	Availability/feasibility	Quality	Comparability over time and space	Relevance to planning	Total
Excess daily all-cause mortality due to heat	2	1	1	1	2	9
Daily all-cause mortality	0	2	2	2	2	8
Daily non-accidental mortality	0	2	2	2	2	8
Daily cardiovascular mortality	0	2	1	2	2	7
Daily respiratory mortality	0	2	1	2	2	7
Neoplasm mortality	0	2	1	2	2	7
Myocardial infarction mortality	0	2	1	2	2	7
Daily mortality (=deaths due to cardiovascular disease + deaths due to respiratory disease + deaths due to all other diseases)	0	2	1	2	2	7
Heat deaths during summer months	1	1	0	1	1	5
Deaths due to heat stroke or heat exhaustion	1	1	0	1	1	5
Deaths due to heat	1	1	1	0	1	5
Hospital admissions (all)	0	1	1	2	2	6
Hospital visits for cardiovascular diseases	0	1	0	1	1	3
Deaths due to cardiovascular and respiratory diseases	0	2	1	1	1	5
Hospital admission excess for electrolyte imbalance, acute renal failure, nephritis, and heat related illnesses	1	1	0	1	1	5
Excess morbidity due to heat (ER visits and hospitalizations during summer months)	1	1	0	1	1	5
Hospital admissions for renal diseases, acute renal failure, and dialysis	0	1	0	1	1	3
Hospital admissions for cardiovascular, cerebrovascular, and respiratory causes	0	1	0	1	1	3
Fire dispatches for heat-related medical events	1	1	0	1	1	5
Ambulance calls for heat-related illnesses	1	1	0	1	1	5
All ED presentations	0	1	1	2	2	6

**Table 4:** Scoring of existing climate change and health indicators for air pollution related health effects

Indicator	Specificity (weighted × 2)	Availability/feasibility	Quality	Comparability over time and space	Relevance to planning	Total
Premature deaths due to PM exposure	1	0	1	1	2	6
Premature deaths due to air pollution (ozone and PM 2.5)	2	0	1	1	2	8
Daily mortality due to ozone	1	0	1	1	2	6
Respiratory/allergic disease and mortality related to increased air pollution and pollens	1	0	1	1	2	6
Daily all-cause mortality	0	2	2	2	2	8
COPD mortality among adult women	0	1	1	1	1	4
Lower respiratory infection mortality among children <5	0	1	1	1	1	4
Neonatal, infant, and elder mortality	0	2	1	1	1	5
Bronchitis: chronic and acute incidence and prevalence	0	0	0	2	2	4
Asthma attack incidence and prevalence	0	1	0	2	1	4
Lower and upper respiratory illness symptom incidence and prevalence	0	1	0	1	2	4
Days of work lost	0	1	0	2	2	5
Moderate or worse asthma status prevalence	0	0	0	2	1	3
Days with restricted activity	0	0	0	2	1	3
Mortality due to smoke inhalation	0	1	0	2	2	5
Asthma incidence	0	2	1	2	2	7
Asthma prevalence	0	2	1	2	2	7
Atopic eczema prevalence	0	1	0	2	2	5
Allergic respiratory disease incidence	0	1	0	2	2	5
Daily non-accidental mortality	0	2	2	2	2	8
Daily respiratory mortality	0	2	1	2	2	7
Daily cardiovascular mortality	0	2	1	2	2	7
Daily non-accidental + respiratory + cardiovascular mortality	0	2	1	2	2	7
Hospital admissions for cardiovascular and respiratory diseases	0	1	0	1	1	3
ER visits for asthma and wheeze	0	1	0	1	1	3
Acute respiratory illness prevalence	0	0	0	2	2	4
Lung CA prevalence	0	2	2	2	1	7
Anti-allergy medication sales	0	1	1	1	2	5

**Table 5:** Scoring of existing climate change and health indicators for extreme weather-related health effects

Indicator	Specificity (weighted × 2)	Availability/feasibility	Quality	Comparability over time and space	Relevance to planning	Total
Disaster mortality	0	2	1	0	2	5
Injuries and deaths due to extreme weather events	1	1	1	0	2	6
Excess accidental and non-accidental deaths	1	1	1	1	2	7
Mortality due to cyclones	0	1	1	0	2	4
Hurricane related deaths	0	1	1	0	2	4
Mortality from flooding	0	1	1	0	2	4
ER visits	0	1	1	2	2	6
Mortality due to wildfire or mudslides	0	1	1	0	2	4
Psychological distress on GHQ12	0	0	1	1	2	4
Infectious and non-infectious diarrhea incidence	0	1	1	0	2	4
Hospital admissions associated with diarrhea	0	1	1	0	1	3

**Table 6:** Scoring of existing climate change and health indicators for water and food contamination-related health effects

Indicator	Specificity (weighted × 2)	Availability/feasibility	Quality	Comparability over time and space	Relevance to planning	Total
Cryptosporidiosis incidence	0	2	1	1	2	6
Samonellosis incidence (and seasonality)	0	2	1	1	2	6
Giardiasis incidence	0	2	1	1	2	6
Enteric protozoa infection incidence	0	1	1	1	2	5
Gastroenteritis incidence	0	1	1	1	2	5
Bacillary dysentery incidence (Shigellosis)	0	2	1	1	2	6
Cholera prevalence	0	2	1	1	0	4

**Table 7:** Scoring of existing climate change and health indicators for vector-borne infectious diseases

Indicator	Specificity (weighted × 2)	Availability/feasibility	Quality	Comparability over time and space	Relevance to planning	Total
Japanese encephalitis incidence	1	2	1	1	0	6
Tick-borne encephalitis incidence	1	2	1	1	0	6
West Nile disease incidence (in humans)	1	2	1	1	2	8
Lyme borreliosis incidence (in humans)	1	2	1	1	2	8
Human cases of Hantavirus	1	2	1	1	0	6
Human cases of Rift valley fever	1	2	1	1	0	6
Dengue fever incidence	1	2	1	1	0	6
Malaria prevalence	1	2	1	1	0	6
Hospital admission for viral pneumonia	0	1	1	1	1	4

**Table 8:** Scoring of existing climate change and health indicators for stratospheric ozone depletion-related health effects

Indicator	Specificity (weighted × 2)	Availability/feasibility	Quality	Comparability over time and space	Relevance to planning	Total
Skin CA incidence	0	2	1	2	2	6
Pemphigus vulgaris incidence	0	1	1	1	1	4

**Table 9:** Scoring of existing climate change and health indicators for other health effects

Indicator	Specificity (weighted × 2)	Availability/feasibility	Quality	Comparability over time and space	Relevance to planning	Total
Preventable deaths from climate change	2	0	0	2	2	8
DALYs lost from climate change	2	0	0	2	2	8
Malnutrition prevalence	0	1	1	2	0	4

**Table 10:** Summary of climate change and health outcome indicator scores

Score	Health effect described	Indicator
9	Temperature extremes	Excess daily all-cause mortality due to heat
8	Temperature extremes and air pollution-related	Daily all-cause mortality
8	Temperature extremes and air pollution-related	Daily non-accidental mortality
8	Air pollution-related	Premature deaths due to air pollution (ozone and PM2.5)
8	Infectious disease	West Nile disease incidence (in humans)
8	Infectious disease	Lyme borreliosis incidence (in humans)
8	All/other	Preventable deaths from climate change
8	All/other	DALY’s lost from climate change
7	Temperature extremes and air pollution-related	Daily respiratory mortality
7	Temperature extremes and air pollution-related	Daily cardiovascular mortality
7	Temperature extremes	Neoplasm mortality
7	Temperature extremes	Myocardial infarction mortality
7	Temperature extremes	Daily mortality calculated as cardiovascular + respiratory + all other disease mortality
7	Air pollution-related	Daily mortality calculated as cardiovascular + respiratory + non-accidental mortality
7	Air pollution-related	Asthma incidence
7	Air pollution-related	Asthma prevalence
7	Extreme weather events	Excess accidental and non-accidental deaths related to extreme weather
6	Temperature extremes	Number of emergency department presentations (all)
6	Temperature extremes	Hospital admissions (all)
6	Air pollution-related	Respiratory/allergic disease and mortality related to increased air pollution and pollens
6	Air pollution-related	Daily mortality due to ozone
6	Air pollution-related	Premature deaths due to PM exposure
6	Extreme weather events	Injuries and deaths due to extreme weather events
6	Extreme weather events	ER visits
6	Food and water contamination	Cryptosporidiosis incidence
6	Food and water contamination	Samonellosis incidence
6	Food and water contamination	Giardiasis incidence
6	Food and water contamination	Shigellosis incidence
6	Infectious disease	Tick-borne encephalitis incidence
6	Infectious disease	Japanese encephalitis incidence
6	Infectious disease	Dengue fever incidence
6	Infectious disease	Malaria prevalence
6	Infectious disease	Human cases of Hantavirus
6	Infectious disease	Human cases of Rift valley fever
6	Stratospheric ozone depletion	Skin cancer incidence
5	Temperature extremes	Heat deaths during summer months
5	Temperature extremes	Deaths due to heat stroke or heat exhaustion
5	Temperature extremes	Excess morbidity due to heat (ER visits and hospitalizations during summer months)
5	Temperature extremes	Deaths due to heat
5	Temperature extremes	Deaths due to cardiovascular and respiratory diseases
5	Temperature extremes	Hospital admission excess for electrolyte imbalance, acute renal failure, nephritis, and heat related illnesses
5	Temperature extremes	Fire dispatches for heat-related medical events
5	Temperature extremes	Ambulance calls for heat-related illnesses
5	Air pollution-related	Lung CA prevalence
5	Air pollution-related	Atopic eczema prevalence
5	Air pollution-related	Allergic respiratory disease incidence
5	Air pollution-related	Anti-allergy medication sales
5	Air pollution-related	Neonatal, infant, and elder mortality
5	Air pollution-related	Days of work lost
5	Air pollution-related	Mortality due to smoke inhalation
5	Extreme weather events	Disaster mortality
5	Food and water contamination	Enteric protozoa infection incidence
5	Food and water contamination	Gastroenteritis incidence
4	Air pollution-related	COPD mortality among adult women
4	Air pollution-related	Lower respiratory infection mortality among children <5
4	Air pollution-related	Acute respiratory illness prevalence
4	Air pollution-related	Bronchitis: chronic and acute incidence and prevalence
4	Air pollution-related	Asthma attack incidence and prevalence
4	Air pollution-related	Lower and upper respiratory illness symptom incidence and prevalence
4	Extreme weather events	Mortality due to cyclones
4	Extreme weather events	Infectious and non-infectious diarrhea incidence (associated with rainfall)
4	Extreme weather events	Psychological distress on General Health Questionnaire 12
4	Extreme weather events	Hurricane-related deaths
4	Extreme weather events	Mortality from flooding
4	Extreme weather events	Mortality due to wildfire or mudslides
4	Food and water contamination	Cholera prevalence
4	Infectious disease	Hospital admission for viral pneumonia
4	Stratospheric ozone depletion	Pemphigus vulgaris incidence
4	All/other	Malnutrition prevalence
3	Temperature extremes	Hospital admissions for renal diseases, acute renal failure, and dialysis
3	Temperature extremes	Hospital admissions for cardiovascular, cerebrovascular, and respiratory causes
3	Temperature extremes	Hospital admissions for cardiovascular diseases
3	Air pollution-related	Hospital admissions for cardiovascular and respiratory diseases
3	Air pollution-related	ER visits for asthma and wheeze
3	Air pollution-related	Moderate or worse asthma status prevalence
3	Air pollution-related	Days with restricted activity
3	Extreme weather events	Hospital admissions associated with diarrhea (associated with rainfall)

The final list of proposed climate change and health outcome indicators for use in Canada includes those with a score of eight or higher based on the ranking criteria. The following eight indicators scored high enough to be included in the final “basket”;

Modeled mortality indicators:

- Excess daily all-cause mortality due to heat.
- Premature deaths due to air pollution (ozone and Particulate Matter (PM) 2.5).
- Preventable deaths from climate change.
- Disability-adjusted life years (DALY’s) lost from climate change.

Non-modeled indicators:

- Daily all-cause mortality (trends associated with heat and air pollution).
- Daily non-accidental mortality (trends associated with heat and air pollution).
- West Nile disease incidence (in humans).
- Lyme borreliosis incidence (in humans).

## Discussion

The final “basket” of eight indicators rated the highest for quantifying health outcomes related to climate change in Canada based on the five evaluation criteria used in this report. Half of the indicators are modeled, which offers both benefits and challenges. Modeled indicators are defined, in the context of this research, as indicators that require statistical calculations and modeling based on collected data. For example, a city may have the data for daily mortality rates and temperature measurements. In order to calculate excess mortality due to heat, modeling on past rates of mortality needs to be performed to determine the expected mortality rates, which can then be analyzed against temperature data to determine the excess mortality due to heat. Modeled indicators are valuable in quantifying the health impacts of climate change because they are better defined in terms of their specificity (i.e., their relationship to climate change), because they can incorporate emerging scientific findings about exposures to climate hazards, and because they are particularly useful in describing projected climate change effects on health. However, modeled indicators are often less comparable than non-modeled indicators and they vary in quality depending on calculation methods; they also vary in quality due to the greater uncertainty associated with necessary assumptions that must be made to calculate them. Also, problematic for public health adaptation applications, the modeled indicators identified here are not currently available in for many communities in Canada. Considering the value of these indicators, this paper makes a strong case for their development.

The non-modeled indicators identified are more readily available for immediate use by Canadian public health authorities. They are particularly valuable in the assessment of climate change-related public health outcomes for local/municipal governments and health units because they are easy to use and analyze. Despite the relative simplicity of the four non-modeled indicators, however, daily all-cause mortality and daily non-accidental mortality due to heat and air pollution require time-series analysis and statistical processing including adjustment for confounders before they can be used for climate change and health studies. For those reasons, daily mortality data may be more suitable for use on an annual basis at provincial/territorial or federal agencies that have greater capacities for statistical support and analysis. At the local level, the most readily available data from the basket of indicators are West Nile and Lyme Disease incidence in humans. Throughout Canada, these diseases are reportable diseases to public health, although issues with underreporting exist (Public Health Agency of Canada 2005).

In addition to the proposed basket of eight indicators, local health units may find others important to use to monitor climate change impacts on health over time or to inform assessments. In Table 10, all 77 indicators rated are listed in descending order based on their individual scores; health authorities may use this list to identify other indicators tailored to their specific needs. For example, a certain health unit may have local data on asthma incidence and prevalence, which rated well and, therefore, would be useful for their jurisdiction. Despite the efforts of this study, rating scores may not be the same for each level of government in varying areas in Canada. We encourage the users to incorporate local knowledge in using this list of indicators.

The indicators involved in this study were complex due to technical and methodological differences. Even though indicators may have similar names in different reports and articles, for instance, various researchers may use different collection and calculation methods. Consequently, two reports may refer to different relationships while referring to the same indicator. At this current stage, we acknowledge that this project was not able to address this issue. In our rankings of quality, much like other criteria, we attempted rankings based on general problems with indicators, relative to each other, rather than attending to methodological distinctions. For example, for mortality indicators due to certain causes, we have rated them lower than mortality indicators not due to specific causes, based on the reasoning that identifying cause of death is often difficult and contains greater room for error than identifying a death without specifying a cause. Each user of the indicators may consider these issues for their jurisdiction and be fully aware of potential difficulties in calculation, definition, and maintenance of the indicators. As indicators are adopted for use, we will understand more clearly what issues are the most prominent and find solutions accordingly.

The multifaceted relationship between climate change, public health, and health indicators adds another layer of complexity to this research. The relationship is complex because most climate-sensitive health impacts have many causal factors in addition to climate and because the causal relationships between climate change and public health can be indirect and non-linear. In some instances there is also a lag time between exposure and response, which can affect certain groups differently and can result in varying degrees of confidence when trying to link climate change and health (McMichael 1995; Ebi 2008; Vineis 2010; Xun et al. 2010; Forsberg et al. 2012). Indentifying useful indicators is also difficult because of the unpredictability of climate change itself. As current scientific findings indicate, the future health impacts from climate change will likely worsen and Canadians may face, in certain regions or communities, new health threats that have not yet been experienced (e.g., exotic disease) (Seguin 2008). Additionally, as the body of research surrounding climate change grows, health effects currently unknown or considered of less concern may receive more attention from researchers. For example, there may be a need to consider both the acute and chronic health outcomes to fully understand the risks facing Canadians. While this paper does not clearly separate the health effects into acute and chronic, we have included indicators that can belong to both categories based on current knowledge. For example, while heat and air pollution related health effects may be immediately visible in the data (acute), it may take a number of years to identify a changing trend in the pattern of infectious diseases such as Lyme borreliosis (chronic). The relationship between climate change and health is dynamic and a rapidly expanding area of science; this current basket of indicators will need to be re-evaluated and modified on a regular basis based on new scientific findings.

Last, the project reported here did not investigate all possible health effects influenced by climate change. Some health outcomes, such as the health effects of harmful algal blooms and Arctic glacial melting, have not been considered through the literature review and rating of indicators. We have limited the scope of this report to the health effects identified in Fig. 1 due to resource restrictions. We were also not able to address special population effects within health outcomes, i.e. vulnerable groups and special populations. Further research can expand the scope of this project to include a larger range of health effects and address the differential health effects of climate change on different population groups.

### Conclusions

In conclusion, in proposing this basket of eight indicators for quantifying climate change health effects, we make two overarching observations. The first is that we currently lack modeled indicators and models for evaluating the health effects of climate change even though modeled indicators are the best at informing action; consequently, we argue that there is a need to develop these indicators. The second main observation regarding the use of these indicators is that each jurisdiction may need to customize the use of them depending on a variety of local factors including resources, data availability, and applicability to the specific group served; each jurisdiction will also need to continuously evaluate and modify their analysis.

In responding to the threats of climate change, this project helps in efforts to monitor, assess, and project human health effects through quantifying such effects, as well as through the evaluation of existing and planned adaptation efforts. This is a promising step for assisting Canadians in climate change adaptation, and the process may also be applicable in other countries. We propose the following research needs as next steps to help further adaption to the effects of this national and global problem:

- modeled indicators in Canada and globally (including extreme heat and air pollution-related excess and premature deaths).
- Streamlined and consistent definitions and calculations for modeled indicators.
- Investigation of other health outcomes from climate change reflecting the changing importance of each health outcome and ;
- expansion of the indicators to include other useful areas such as differential effects of climate change on different groups (vulnerabilities and adaptive capacity).

## Electronic supplementary material

Below is the link to the electronic supplementary material.

Supplementary material 1 (PDF 200 kb)
