# Supplementary material for: Development of key indicators to quantify the health impacts of climate change on Canadians
Source: Int J Public Health. 2013 Jul 30;58(5):765–75. doi: 10.1007/s00038-013-0499-5 (PMC3907783; doi:10.1007/s00038-013-0499-5)
Supplement: Supplementary file 1 — Supplementary material 1 (PDF 200 kb) [file 38_2013_499_MOESM1_ESM.pdf]

Online Resource: Supplemental Materials

Development of Key Indicators to Quantify the Health Impacts of Climate Change on Canadians

June J. Cheng MD MPH[1,2*] and Peter Berry PhD[2]

[1] Public Health and Preventive Medicine Residency, Department of Clinical Epidemiology & Biostatistics, McMaster University, Hamilton, Ontario, Canada

Email: june.cheng@medportal.ca

[2] Climate Change and Health Office, Health Canada, Ottawa, Ontario, Canada

* Corresponding Author

Table of Contents

Table S1. Existing climate change and health indicators due to temperature extremes Pg. 2

Table S2. Existing climate change and health indicators due to air pollution Pg. 4

Table S3. Existing climate change and health indicators due to extreme weather events Pg. 5

Table S4. Existing climate change and health indicators due to food and water contamination Pg. 6

Table S5. Existing climate change and health indicators due to infectious diseases Pg. 7

Table S6. Existing climate change and health indicators due to stratospheric ozone depletion Pg. 8

Table S7. Other existing climate change and health indicators Pg. 9

Table S8. Potential criteria for evaluating proposed climate change and health indicators Pg. 10

Supplemental Materials References Pg. 12

Supplemental Material, Table S1. Existing climate change and health indicators due to temperature extremes

Indicator	Used By
Excess daily all-cause mortality due to heat	Armstrong et al. 2011; Baccini et al. 2009; Ballester et al. 2011; Barnett et al. 2012; Davis et al. 2003a; Davis et al. 2003b; Dessai 2003; Doherty et al. 2009; Donaldson et al. 2003; Gosling et al. 2007; Green et al. 2012; Hoshiko et al. 2010; Kalkstein 1993; Kalkstein 1991; Kalkstein and Greene 1997; Knowlton et al. 2007; Michelozzi et al. 2010; Robine et al. 2008; Dalbokova et al. 2009; WHO Regional Office for Europe 2011; English et al. 2009; California EPA 2009; Smoyer-Tomic and Rainham 2001
Daily all-cause mortality	Burkart et al. 2011; D'Ippoliti et al. 2010; Doyon et al. 2008; Fouillet et al. 2007; Gabriel and Endlicher 2011; Huynen et al. 2001; Iniguez et al. 2010; Kim et al. 2006; Laaidi et al. 2006; Langford and Bentham 1995; Martens 1998; McMichael et al. 2008; O'Neill et al. 2005; Rocklov and Forsberg 2010; Williams et al. 2012; Yu et al. 2011; Yu et al. 2012
Daily non-accidental mortality	Kan et al. 2007; Martin et al. 2011; Montero et al. 2012; O'Neill et al. 2003; Pengelly et al. 2007; Revich and Shaposhnikov 2010; Tong et al. 2010
Daily cardiovascular mortality	Burkart et al. 2011; Huynen et al. 2001; Kan et al. 2007; Martens 1998; Revich and Shaposhnivok 2010; Tong et al. 2010; Yu et al. 2011
Daily respiratory mortality	Huynen et al. 2001; Kan et al. 2007; Martens 1998
Neoplasm mortality	Huynen et al. 2001
Myocardial infarction mortality	Crawford et al. 2003
Daily mortality = deaths due to cardiovascular disease + deaths due to respiratory disease + deaths due to all other diseases	Curriero et al. 2002
Heat deaths during summer months	English et al. no date
Deaths due to heat stroke or heat exhaustion	National Environmental Public Health Tracking Program 2012
Deaths due to heat	McGeehin and Mirabelli 2001
Hospital admissions (all)	McGeehin and Mirabelli 2001
Hsopital visits for cardiovascular diseases	McGeehin and Mirabelli 2001
Deaths due to cardiovascular and respiratory diseases	McGeehin and Mirabelli 2001
Hospital admission excess for electrolyte imbalance, acute renal failure, nephritis, and heat related illnesses	Knowlton et al. 2011
Excess morbidity due to heat (emergency room visits and hospitalizations during summer months)	English et al. 2009; California EPA 2009
Hospital admissions for renal diseases, acute renal failure, and dialysis	Hansen et al. 2008
Hospital admissions for cardiovascular, cerebrovascular,	Michelozzi et al. 2009

and respiratory causes	
Fire dispatches for heat-related medical events	Golden et al. 2008
Ambulance calls for heat-related illness	Bassil et al. 2011
ED presentations (all)	Williams et al. 2012; McGeehin and Mirabelli 2001; Knowlton et al. 2011

Supplemental Material, Table S2. Existing climate change and health indicators due to air pollution

Indicator	Used By
Avoidable deaths due to PM exposure	Anonymous 1997
Premature deaths due to air pollution (ozone and PM2.5)	Tagaris et al. 2010; Tagaris et al. 2009
COPD mortality among adult women	Bailis et al. 2005
Lower respiratory infection mortality among children <5	Bailis et al. 2005
Neonatal, infant, and elder mortality	Cifuentes et al. 2001
Bronchitis: chronic and acute incidence and prevalence	Cifuentes et al. 2001
Asthma attack incidence and prevalence	Cifuentes et al. 2001
Lower & upper respiratory illness symptom incidence and prevalence	Cifuentes et al. 2001
Days of work lost	Cifuentes et al. 2001
Moderate or worse asthma status prevalence	Cifuentes et al. 2001
Days with restricted activity	Cifuentes et al. 2001
Mortality due to smoke inhalation	Knowlton et al. 2011
Asthma incidence	Beggs and Bambrick 2005; D'Amato et al. 2010; Shea et al. 2008
Asthma prevalence	Beggs and Bambrick 2005; Shea et al. 2008; Weiland et al. 2004; McMichael et al. 2004
Atopic Eczema prevalence	Weiland et al. 2004
Allergic respiratory disease incidence	D'Amato et al. 2010
Daily non-accidental mortality	Chang et al. 2010; Johnston et al. 2011; Park et al. 2011
Daily respiratory mortality	Johnston et al. 2011; Park et al. 2011
Daily cardiovascular mortality	Johnston et al. 2011; Park et al. 2011
Daily non-accidental + respiratory + cardiovascular mortality	Yi et al. 2010
Hospital admissions for cardiovascular and respiratory diseases	Yi et al. 2010; Cifuentes et al. 2001; Knowlton et al. 2011
ER visits for asthma and wheeze	Darrow et al. 2012; Sheffield et al. 2011; Cifuentes et al. 2001; Knowlton et al. 2011
Daily mortality due to ozone	Doherty et al. 2009; Knowlton et al. 2004; West et al. 2006; Knowlton et al. 2011
Cardio-respiratory mortality	WHO Regional Office for Europe 2011; McMichael et al. 2004
Acute respiratory illnesses	McMichael et al. 2004
Lung CA	McMichael et al. 2004
Anti-allergy medication sales	WHO Regional Office for Europe 2011
All-cause mortality	Kalkstein 1991
Respiratory/allergic disease and mortality related to increased air pollution and pollens	English et al. 2009

Supplemental Material, Table S3. Existing climate change and health indicators due to extreme weather events

**Indicator**	**Used By**
Disaster Mortality	Seguin 2008; Greenough et al. 2001; Myung and Jang 2011; English et al. 2009
Injuries and deaths due to extreme weather events	English et al. no date
Excess accidental and non-accidental deaths	Anderson and Bell 2012
Mortality during cyclones	Haque et al. 2012
Hurricane related deaths	Knowlton et al. 2011
Mortality from flooding	Knowlton et al. 2011
ER visits	Knowlton et al. 2011
Mortality due to wildfire or mudslides	Knowlton et al. 2011
Psychological Distress (>4 on 12 item General Health Questionnaire (GHQ12))	Reacher et al. 2004
Hospital admissions associated with diarrhea	Chou et al. 2010
Infectious and non-infectious diarrhea incidence	Chou et al. 2010

Supplemental Material, Table S4. Existing climate change and health indicators due to food and water contamination

Indicator	Used By
Cryptosporidiosis incidence	Britton et al. 2010; Hu et al. 2010; WHO Regional Office for Europe 2011; Dalbokova et al. 2009
Samonellosis incidence and seasonality	WHO Regional Office for Europe 2011; Dalbokova et al. 2009
Giardiasis incidence	Britton et al. 2010
Enteric protozoa infection surveillance	Fletcher et al. 2012
Gastroenteritis cases	Hall et al. 2011
Bacillary dysentery cases	Zhang et al. 2007b; Zhang et al. 2008
Cholera prevalence	Reyburn et al. 2011

Supplemental Material, Table S5. Existing climate change and health indicators due to infectious diseases

Indicator	Used By
Incidence of Japanese encephalitis	Bi et al. 2007
Tick-Borne Encephalitis incidence (in humans)	Daniel et al. 2011
West Nile Virus disease incidence (in humans)	DeGroote et al. 2008; Wang et al. 2010; English et al. 2009; English et al. no date; Knowlton et al. 2011
Hospital admission for viral pneumonia	Ebi et al. 2001
Dengue incidence	Russell et al. 2009; English et al. no date
Human cases of hantavirus	English et al., no date
Human cases of Valley fever	English et al., no date
Malaria Prevalence	Martens 1995; Gething et al. 2010
Incidence lyme borreliosis	Odgen et al. 2008; WHO Regional Office for Europe 2011; Dalbokova et al. 2009; English et al. 2009; English et al. no date

Supplemental Material, Table S6. Existing climate change and health indicators due to stratospheric ozone depletion

Indicator	Used By
Skin Cancer Incidence	DARA 2012
Pemphigus vulgaris incidence	Kyriakis et al. 1995

Supplemental Material, Table S7. Other existing climate change and health indicators

Indicator	Used By
Preventable deaths from climate change	Cole 2009
DALYs lost from climate change	Costello et al. 2009; Zhang et al. 2007a
Malnutrition, food insecurity	Darnton-Hill and Cogill 2010

Supplemental Material, Table S8. Potential criteria for evaluating proposed climate change and health indicators

Framework/Organization	Criteria
Demographic & Health Indicators selected by Statistics Canada<br>Peron and Strohmenger 1985	Availability or feasibility<br>Stability of sources and objectivity of calculation<br>Intelligibility
Association of Public Health Epidemiologists of Ontario, no date	Applicable to outcomes from Ontario Public Health Standards<br>Useful<br>Meaningful<br>Relevant to health status<br>Applicable data available to all or most local public health units<br>Core indicator important to public health<br>Decisions by consensus
Ontario District Health Councils, no date	Validity<br>Feasibility<br>Understandable<br>Relevance to planning<br>Comparability over time and place<br>Amenable to action<br>Quality acceptable
NHS Institute for Innovation and Improvement 2008	Important (measures important issue)<br>Valid<br>Feasible/possible<br>Reliable
National Collaborating Centre for Methods and Tools 2010	Evaluation<br>Validity<br>Reliability<br>Methodological rating
United States Environmental Protection Agency 2012	Usefulness<br>Objectivity<br>Data quality<br>Transparency<br>Ability to show a meaningful trend<br>Relevance to climate change
National Research Council Board on Atmospheric Sciences and Climate 2010	Direct<br>Significant<br>Dominant<br>Measureable<br>Historical<br>Well-documented
California EPA 2009	Availability
English et al. No date	Data quality/completeness<br>Availability<br>Temporality<br>Sensitivity<br>Overall-readiness reported from previous 4 characteristics
English et al. 2009	Availability<br>Temporality<br>Completeness
Wardekker et al. 2012	Level of precision rated by experts on a variety of

	climate change and health indicators for the Netherlands
Hambling et al. 2012	Credible<br>Specific<br>Actionable<br>Sensitive to changes in climate and less sensitive to alternate explanations<br>Relevant to an issue of policy<br>Sustainable<br>Consistent and comparable over time and space<br>Scalable<br>Robust<br>Unbiased and representative<br>Explicit<br>Accurate<br>Understandable, applicable, acceptable to stakeholders potential users<br>Measurable<br>Cost-effective<br>Selective<br>Available in a timely manner

Supplemental Materials References

Anonymous (1997) Short-term improvements in public health from global-climate policies on fossil-fuel combustion: An interim report. Working group on public health and fossil-fuel combustion. Lancet 350(9088):1341-1349

Anderson GB, Bell ML (2012) Lights out: Impact of the august 2003 power outage on mortality in New York, NY. Epidemiol 23(2):189-193

Armstrong BG, Chalabi Z, Fenn B, Hajat S, Kovats S, Milojevic A et al. (2011) Association of mortality with high temperatures in a temperate climate: England and Wales. J Epidemiol & Community Health 65(4):340-345

Association of Public Health Epidemiologists of Ontario (No date) The Core Indicators for Public Health in Ontario. http://www.apheo.ca/resources/indicators/APHEO_Core%20Indicators%20brochure.pdf. Accessed January 4, 2013

Baccini M, Kosatsky T, Analitis A, Anderson HR, D'Ovidio M, Menne B et al. (2011) Impact of heat on mortality in 15 European cities: Attributable deaths under different weather scenarios. J Epidemiol & Community Health 65(1):64-70

Bailis R, Ezzati M, Kammen DM (2005) Mortality and greenhouse gas impacts of biomass and petroleum energy futures in Africa. Science 308(5718):98-103

Ballester J, Robine JM, Herrmann FR, Rodo X (2011) Long-term projections and acclimatization scenarios of temperature-related mortality in Europe. Nature commun 2:358

Barnett AG, Hajat S, Gasparrini A, Rocklov J (2012) Cold and heat waves in the United States. Environ Res 112:218-224

Bassil KL, Cole DC, Moineddin R, Lou W, Craig AM, Schwartz B et al. (2011) The relationship between temperature and ambulance response calls for heat-related illness in Toronto, Ontario, 2005. J Epidemiol & Community Health 65(9):829-831

Beggs PJ, Bambrick HJ (2005) Is the global rise of asthma an early impact of anthropogenic climate change? Environ Health Perspect 113(8):915-919

Bi P, Zhang Y, Parton KA (2007) Weather variables and Japanese encephalitis in the metropolitan area of Jinan City, China. J Infect 55(6):551-556

Britton E, Hales S, Venugopal K, Baker MG (2010) The impact of climate variability and change on Cryptosporidiosis and Giardiasis rates in New Zealand. J Water & Health 8(3):561-571

Burkart K, Schneider A, Breitner S, Khan MH, Kramer A, Endlicher W (2011) The effect of atmospheric thermal conditions and urban thermal pollution on all-cause and cardiovascular mortality in Bangladesh. Environ Pollution 159(8-9):2035-2043

California Environmental Protection Agency (2009) Indicators of Climate Change in California Report Summary. http://www.oehha.ca.gov/multimedia/epic/pdf/ClimateSummary.pdf. Accessed January 3, 2013

Chang HH, Zhou J, Fuentes M (2010) Impact of climate change on ambient ozone level and mortality in southeastern United States. Int J Environ Res & Public Health 7(7):2866-2880

Chou WC, Wu JL, Wang YC, Huang H, Sung FC, Chuang CY (2010) Modeling the impact of climate variability on diarrhea-associated diseases in Taiwan (1996-2007). Sci Total Environ 409(1):43-51

Cifuentes L, Borja-Aburto VH, Gouveia N, Thurston G, Davis DL (2001) Assessing the health benefits of urban air pollution reductions associated with climate change mitigation (2000-2020): Santiago, Sao Paulo, Mexico City, and New York City. Environ Health Perspect 109(Suppl 3):419-425

Cole A (2009) Annual deaths from climate change could reach half a million by 2030. BMJ 338:b2227

Costello A, Abbas M, Allen A, Ball S, Bell S, Bellamy R et al. (2009) Managing the health effects of climate change: Lancet and University College London Institute for Global Health Commission. Lancet 373(9676):1693-1733

Crawford VL, McCann M, Stout RW (2003) Changes in seasonal deaths from myocardial infarction. QJM 96(1):45-52

Curriero FC, Heiner KS, Samet JM, Zeger SL, Strug L, Patz JA (2002) Temperature and mortality in 11 cities of the eastern United States. Am J Epidemiol 155(1):80-87

Dalbokova D, Krzyzanowski M, Menne B, Matthies F (2009) Development of Health-Relevant Indicators of Climate Change for Europe. Epidemiol 20(6):S57 Abstracts: ISEE 21st Annual Conference, Dublin, Ireland, August 25-29, 2009: Oral PresentationsDalbokova as cited in English et al. no date.

D'Amato G, Cecchi L, D'Amato M, Liccardi G (2010) Urban air pollution and climate change as environmental risk factors of respiratory allergy: An update. J Investig Allerg & Clin Immunol 20(2):95-102

Daniel M, Benes C, Danielova V, Kriz B (2011) Sixty years of research of tick-borne encephalitis--a basis of the current knowledge of the epidemiological situation in central Europe. Epidemiol, Mikrobiol, Imunol 60(4):135-155

DARA (2012) Climate Vulnerability Monitor: a guide to the cold calculus of a hot planet. 2nd edition. Geneva:DARA. http://daraint.org/climate-vulnerability-monitor/climate-vulnerability-monitor-2012/report/. Accessed January 13, 2013

Darnton-Hill I, Cogill B (2010) Maternal and young child nutrition adversely affected by external shocks such as increasing global food prices. J Nutr 140(1):162S-9S

Darrow LA, Hess J, Rogers CA, Tolbert PE, Klein M, Sarnat SE (2012) Ambient pollen concentrations and emergency department visits for asthma and wheeze. J Allerg & Clin Immunol 130(3):630-638.e4

Davis RE, Knappenberger PC, Michaels PJ, Novicoff WM (2003) Changing heat-related mortality in the United States. Environ Health Perspect 111(14):1712-1718

Davis RE, Knappenberger PC, Novicoff WM, Michaels PJ (2003) Decadal changes in summer mortality in U.S. cities. Int J Biometeorol 47(3):166-175

DeGroote JP, Sugumaran R, Brend SM, Tucker BJ, Bartholomay LC (2008) Landscape, demographic, entomological, and climatic associations with human disease incidence of West Nile virus in the state of Iowa, USA. Int J Health Geogr 7:19

Dessai S (2002) Heat stress and mortality in Lisbon Part I. model construction and validation. Int J Biometerolol 47(6):6-12

D'Ippoliti D, Michelozzi P, Marino C, de'Donato F, Menne B, Katsouyanni K et al. (2010) The impact of heat waves on mortality in 9 European cities: Results from the EuroHEAT project. Environ Health 9:37

Doherty RM, Heal MR, Wilkinson P, Pattenden S, Vieno M, Armstrong B et al. (2009) Current and future climate- and air pollution-mediated impacts on human health. Environ Health 8(Suppl 1):S8

Donaldson GC, Keatinge WR, Nayha S (2003) Changes in summer temperature and heat-related mortality since 1971 in North Carolina, South Finland, and Southeast England. Environ Res 91(1):1-7

Doyon B, Belanger D, Gosselin P (2008) The potential impact of climate change on annual and seasonal mortality for three cities in Quebec, Canada. Int J Health Geogr 7:23

Ebi KL, Exuzides KA, Lau E, Kelsh M, Barnston A (2001) Association of normal weather periods and El Nino events with hospitalization for viral pneumonia in females: California, 1983-1998. Am J Public Health 91(8):1200-1208

English PB, Sinclair AH, Ross Z, Anderson H, Boothe V, Davis C et al. (2009) Environmental health indicators of climate change for the United States: Findings from the state environmental health indicator collaborative. Environ Health Perspect 117(11):1673-1681

English P, Green M, Shaw S, Anderson H, Malecki K, Givens M, Christenson M, Patz J, Lin S (No date) Discussion paper for National Climate Assessment. Environmental Health Indicators for Cliamte Change. As part of the Climate Change Indicators Subcommittee of The Council of State and Territorial Epidemiologists.

Fletcher SM, Stark D, Harkness J, Ellis J (2012) Enteric protozoa in the developed world: A public health perspective. Clin Microbiol Rev 25(3):420-449

Fouillet A, Rey G, Jougla E, Frayssinet P, Bessemoulin P, Hemon D (2007) A predictive model relating daily fluctuations in summer temperatures and mortality rates. BMC Public Health 7:114

Gabriel KM, Endlicher WR (2011) Urban and rural mortality rates during heat waves in Berlin and Brandenburg, Germany. Environ Pollut 159(8-9):2044-2050

Gething PW, Smith DL, Patil AP, Tatem AJ, Snow RW, Hay SI (2010) Climate change and the global malaria recession. Nature 465(7296):342-345

Golden JS, Hartz D, Brazel A, Luber G, Phelan P (2008) A biometeorology study of climate and heat-related morbidity in Phoenix from 2001 to 2006. Int J Biometeorol 52(6):471-480

Gosling SN, McGregor GR, Paldy A (2007) Climate change and heat-related mortality in six cities part 1: Model construction and validation. Int J Biometeorol 51(6):525-540

Green HK, Andrews NJ, Bickler G, Pebody RG (2012) Rapid estimation of excess mortality: Nowcasting during the heatwave alert in England and Wales in june 2011. J Epidemiol & Community Health 66(10):866-868

Greenough G, McGeehin M, Bernard SM, Trtanj J, Riad J, Engelberg D (2001) The potential impacts of climate variability and change on health impacts of extreme weather events in the United States. Environ Health Perspect 109(Suppl 2):191-198

Hall GV, Hanigan IC, Dear KB, Vally H (2011) The influence of weather on community gastroenteritis in Australia. Epidemiol & Infect 139(6):927-936

Hambling T, Weinstein P, Slaney D (2011) A review of frameworks for developing environmental health indicators for climate change and health. Int J Environ Res & Public Health 8(7):2854-2875

Hansen AL, Bi P, Ryan P, Nitschke M, Pisaniello D, Tucker G (2008) The effect of heat waves on hospital admissions for renal disease in a temperate city of Australia. Int J Epidemiol 37(6):1359-1365

Haque U, Hashizume M, Kolivras KN, Overgaard HJ, Das B, Yamamoto T (2012) Reduced death rates from cyclones in Bangladesh: What more needs to be done?. Bull World Health Organ 90(2):150-156

Hoshiko S, English P, Smith D, Trent R (2010) A simple method for estimating excess mortality due to heat waves, as applied to the 2006 California heat wave. Int J Public Health 55(2):133-137

Huynen MM, Martens P, Schram D, Weijenberg MP, Kunst AE (2001) The impact of heat waves and cold spells on mortality rates in the Dutch population. Environ Health Perspect 109(5):463-470

Iniguez C, Ballester F, Ferrandiz J, Perez-Hoyos S, Saez M, Lopez A et al. (2010) Relation between temperature and mortality in thirteen Spanish cities. Int J Environ Res & Public Health 7(8):3196-3210

Johnston F, Hanigan I, Henderson S, Morgan G, Bowman D (2011) Extreme air pollution events from bushfires and dust storms and their association with mortality in Sydney, Australia 1994-2007. Environ Res 111(6):811-816

Kalkstein LS (1991) A new approach to evaluate the impact of climate on human mortality. Environ Health Perspect 96:145-150

Kalkstein LS (1993) Health and climate change. Direct impacts in cities. Lancet 342(8884):1397-1399

Kalkstein LS, Greene JS (1997) An evaluation of climate/mortality relationships in large U.S. cities and the possible impacts of a climate change. Environ Health Perspect 105(1):84-93

Kan H, London SJ, Chen H, Song G, Chen G, Jiang L et al. (2007) Diurnal temperature range and daily mortality in Shanghai, China. Environ Res 103(3):424-431

Kim H, Ha JS, Park J (2006) High temperature, heat index, and mortality in 6 major cities in South Korea. Arch Environ & Occup Health 61(6):265-270

Knowlton K, Lynn B, Goldberg RA, Rosenzweig C, Hogrefe C, Rosenthal JK et al. (2007) Projecting heat-related mortality impacts under a changing climate in the New York City region. Am J Public Health 97(11):2028-2034

Knowlton K, Rosenthal JE, Hogrefe C, Lynn B, Gaffin S, Goldberg R et al. (2004) Assessing ozone-related health impacts under a changing climate. Environ Health Perspect 112(15):1557-1563

Knowlton K, Rotkin-Ellman M, Geballe L, Max W, Solomon GM (2011) Six climate change-related events in the United States accounted for about $14 billion in lost lives and health costs. Health Aff 30(11):2167-2176

Kyriakis KP, Vareltzidis AG, Tosca AD (1995) Environmental factors influencing the biologic behavior of patterns of pemphigus vulgaris: Epidemiologic approach. Int J Dermatol 34(3):181-185

Laaidi M, Laaidi K, Besancenot JP (2006) Temperature-related mortality in france, a comparison between regions with different climates from the perspective of global warming. Int J Biometeorol 51(2):145-153

Langford IH, Bentham G (1995) The potential effects of climate change on winter mortality in England and Wales. Int J Biometeorol 38(3):141-147

Martens WJ (1995) Climate change and malaria: Exploring the risks. Med & War 11(4):202-213

Martens WJ (1998) Climate change, thermal stress and mortality changes. Soc Sci Med 46(3):331-344

Martin SL, Cakmak S, Hebbern CA, Avramescu ML, Tremblay N (2012) Climate change and future temperature-related mortality in 15 Canadian cities. Int J Biometeorol 56(4):605-619

McGeehin MA, Mirabelli M (2001) The potential impacts of climate variability and change on temperature-related morbidity and mortality in the United States. Environ Health Perspect 109(Suppl 2):185-189

McMichael AJ, Campbell-Lendrum D, Kovats S, Edwards S, Wilkinson P, Wilson T, Nicholls R, Hales S, Tanser F, Le Sueur D, Schlesinger M, Andronova N (2004) Chapter 20 Global Climate Change. In: Comparative Quantification of Health Risks, Vol 2. Geneva:WHO, 1543-1650

McMichael AJ, Wilkinson P, Kovats RS, Pattenden S, Hajat S, Armstrong B et al. (2008) International study of temperature, heat and urban mortality: The 'ISOTHURM' project. Int J Epidemiol 37(5):1121-1131

Michelozzi P, Accetta G, De Sario M, D'Ippoliti D, Marino C, Baccini M et al. (2009) High temperature and hospitalizations for cardiovascular and respiratory causes in 12 European cities. Am J Resp & Critical Care Med 179(5):383-389

Michelozzi P, de'Donato FK, Bargagli AM, D'Ippoliti D, De Sario M, Marino C et al. (2010) Surveillance of summer mortality and preparedness to reduce the health impact of heat waves in Italy. Int J Environ Res & Public Health 7(5):2256-2273

Montero JC, Miron IJ, Criado-Alvarez JJ, Linares C, Diaz J (2012) Influence of local factors in the relationship between mortality and heat waves: Castile-la mancha (1975-2003). Sci Total Environ 414:73-80

Myung HN, Jang JY (2011) Causes of death and demographic characteristics of victims of meteorological disasters in Korea from 1990 to 2008. Environ Health 10:82

National Collaborating Centre for Methods and Tools (2010) Guidelines for selecting and using indicators. Hamilton, ON: McMaster University. http://www.nccmt.ca/registry/view/eng/73.html. Accessed January 3, 2013

National Environmental Public Health Tracking Program (2012) CDC Query and Results. http://ephtracking.cdc.gov/showLocationLanding.action. Accessed March 6, 2013

National Research Council Board on Atmospheric Sciences and Climate (2010) Monitoring Climate Change Impacts: Metrics at the Intersection of the Human and Earth Systems. Washington, DC:The National Academies Press. http://www.nap.edu/openbook.php?record_id=12965&page=R1. Accessed January 3, 2013

NHS Institute for Innovation and Improvement (2008) The Good Indicators Guide: Understanding how to use and choose indicators. http://www.apho.org.uk/resource/item.aspx?RID=44584. Accessed January 4, 2013

Ogden NH, Lindsay LR, Morshed M, Sockett PN, Artsob H (2008) The rising challenge of lyme borreliosis in Canada. Can Commun Dis Rep 34(1):1-19

O'Neill MS, Hajat S, Zanobetti A, Ramirez-Aguilar M, Schwartz J (2005) Impact of control for air pollution and respiratory epidemics on the estimated associations of temperature and daily mortality. Int J Biometeorol 50(2):121-129

O'Neill MS, Zanobetti A, Schwartz J (2003) Modifiers of the temperature and mortality association in seven US cities. Am J Epidemiol 157(12):1074-1082

Ontario District Health Councils Local Health System Monitoring Technical Working Group (No date) Process Guide for Health Indicator Selection. http://www.ontla.on.ca/library/repository/mon/24001/298297.pdf. Assessed 15 December 2012

Park AK, Hong YC, Kim H (2011) Effect of changes in season and temperature on mortality associated with air pollution in Seoul, Korea. J Epidemiol & Community Health 65(4):368-375

Pengelly LD, Campbell ME, Cheng CS, Fu C, Gingrich SE, Macfarlane R (2007) Anatomy of heat waves and mortality in Toronto: Lessons for public health protection. Can J Public Health 98(5):364-368

Peron Y, Strohmenger C (1985) Demographic and Health Indicators: Presentation and Interpretation. Catalogue No. 82-543. Ottawa:Statistics Canada

Reacher M, McKenzie K, Lane C, Nichols T, Kedge I, Iversen A et al. (2004) Health impacts of flooding in Lewes: A comparison of reported gastrointestinal and other illness and mental health in flooded and non-flooded households. Commun Dis & Public Health 7(1):39-46

Revich BA, Shaposhnikov DA (2010) Extreme temperature episodes and mortality in Yakutsk, East Siberia. Rural & Remote Health 10(2):1338

Reyburn R, Kim DR, Emch M, Khatib A, von Seidlein L, Ali M (2011) Climate variability and the outbreaks of cholera in Zanzibar, East Africa: A time series analysis. Am J Trop Med & Hyg 84(6):862-869

Robine JM, Cheung SL, Le Roy S, Van Oyen H, Griffiths C, Michel JP et al. (2008) Death toll exceeded 70,000 in Europe during the summer of 2003. Comptes Rendus Biologies 331(2):171-178

Rocklov J, Forsberg B (2010) The effect of high ambient temperature on the elderly population in three regions of Sweden. Int J Environ Res & Public Health 7(6):2607-2619

Russell RC, Currie BJ, Lindsay MD, Mackenzie JS, Ritchie SA, Whelan PI (2009) Dengue and climate change in Australia: Predictions for the future should incorporate knowledge from the past. Med J Aust 190(5):265-268

Seguin J, ed. (2008) Human Health in a Changing Climate: A Canadian Assessment of Vulnerabilities and Adaptive Capacity. Ottawa:Health Canada

Shea KM, Truckner RT, Weber RW, Peden DB (2008) Climate change and allergic disease. J Allerg & Clin Immunol 122(3):443-453

Sheffield PE, Knowlton K, Carr JL, Kinney PL (2011) Modeling of regional climate change effects on ground-level ozone and childhood asthma. Am J Prev Med 41(3):251-257

Smoyer-Tomic KE, Rainham DG (2001) Beating the heat: Development and evaluation of a Canadian hot weather health-response plan. Environ Health Perspect 109(12):1241-1248

Tagaris E, Liao KJ, Delucia AJ, Deck L, Amar P, Russell AG (2009) Potential impact of climate change on air pollution-related human health effects. Environ Sci Technol 43(13):4979-4988

Tagaris E, Liao KJ, DeLucia AJ, Deck L, Amar P, Russell AG (2010) Sensitivity of air pollution-induced premature mortality to precursor emissions under the influence of climate change. Int J Environ Res & Public Health 7(5):2222-2237

Tong S, Ren C, Becker N (2010) Excess deaths during the 2004 heatwave in Brisbane, Australia. Int J Biometeorol 54(4):393-400

United States Environmental Protection Agency (2012) Climate Change Indicators in the United States 2012. Washington, DC:EPA. http://www.epa.gov/climatechange/pdfs/climateindicators-full-2012.pdf. Accessed 28 December 2012

Wang G, Minnis RB, Belant JL, Wax CL (2010) Dry weather induces outbreaks of human West Nile virus infections. BMC Infectious Diseases 10:38

Wardekker JA, de Jong A, van Bree L, Turkenburg WC, van der Sluijs JP (2012) Health risks of climate change: An assessment of uncertainties and its implications for adaptation policies. Environ Health 11:67

Weiland SK, Husing A, Strachan DP, Rzehak P, Pearce N, ISAAC Phase One Study G (2004) Climate and the prevalence of symptoms of asthma, allergic rhinitis, and atopic eczema in children. Occup & Environ Med 61(7):609-615

West JJ, Fiore AM, Horowitz LW, Mauzerall DL (2006) Global health benefits of mitigating ozone pollution with methane emission controls. Proc Natl Acad Sci U S A 103(11):3988-3993

WHO Regional Office for Europe. (2011) Tools for the monitoring of Parma Conference commitments: report of a meeting Bonn, Germany 25-26 November 2010. Copenhagen:WHO

Williams S, Nitschke M, Weinstein P, Pisaniello DL, Parton KA, Bi P (2012) The impact of summer temperatures and heatwaves on mortality and morbidity in Perth, Australia 1994-2008. Environ Int 40:33-38

Yi O, Hong YC, Kim H (2010) Seasonal effect of PM(10) concentrations on mortality and morbidity in Seoul, Korea: A temperature-matched case-crossover analysis. Environ Res 110(1):89-95

Yu W, Mengersen K, Hu W, Guo Y, Pan X, Tong S (2011) Assessing the relationship between global warming and mortality: Lag effects of temperature fluctuations by age and mortality categories. Environ Pollut 159(7):1789-1793

Yu W, Mengersen K, Wang X, Ye X, Guo Y, Pan X et al. (2012) Daily average temperature and mortality among the elderly: A meta-analysis and systematic review of epidemiological evidence. Int J Biometeorol 56(4):569-581

Zhang Y, Bi P, Hiller JE (2007) Climate change and disability-adjusted life years. J Environ Health 70(3):32-36

Zhang Y, Bi P, Hiller JE (2008) Weather and the transmission of bacillary dysentery in Jinan, Northern China: A time-series analysis. Public Health Rep 123(1):61-66

Zhang Y, Bi P, Hiller JE, Sun Y, Ryan P (2007) Climate variations and bacillary dysentery in northern and southern cities of China. J Infect 55(2):194-200
